# Pilot Test of Mopati, a Multi-Level Adherence Intervention for People Living with HIV and Their Treatment Partners in Botswana

**DOI:** 10.1007/s12529-023-10233-7

**Published:** 2023-11-13

**Authors:** Laura M. Bogart, Nthabiseng Phaladze, Keonayang Kgotlaetsile, David J. Klein, Kathy Goggin, Mosepele Mosepele

**Affiliations:** 1 RAND Corporation, 1776 Main Street, P.O. Box 2138, Santa Monica, CA 90407-2138 USA; 2 Charles R. Drew University of Medicine and Science, Los Angeles, CA USA; 3 University of Botswana, Gaborone, Botswana; 4 Health Services and Outcomes Research, Children’s Mercy Kansas City, Kansas City, MO USA; 5 Kansas City Schools of Medicine and Pharmacy, University of Missouri, Kansas City, MO USA; 6 Botswana-Harvard AIDS Institute Partnership, Gaborone, Botswana

**Keywords:** Adherence, Antiretroviral therapy, Botswana, HIV, Motivational interviewing, Psychosocial support

## Abstract

**Background:**

Low-cost, scalable strategies are necessary to reach the UNAIDS 2030 target of ending HIV as a public health threat. Use of treatment partners, informal caregivers selected by people living with HIV to support antiretroviral therapy adherence, is one such strategy that is included in many countries’ HIV guidelines, including Botswana, a country with high HIV prevalence.

**Method:**

From June 2021 to June 2022, we pilot tested a clinic-based treatment partner intervention (“Mopati”), including standardized language for providers to guide patients on treatment partner selection and workshops to train treatment partners on providing non-directive support to patients using a non-confrontational, non-judgmental approach. Sixty unsuppressed patients (30 per clinic) and 45 treatment partners (17 intervention, 28 control) were recruited from an intervention–control clinic matched-pair in Gaborone, Botswana.

**Results:**

Mopati had medium-to-large effects on increasing patients’ adherence, adherence self-efficacy, intrinsic adherence motivation, and perceived non-directive support from treatment partners, and decreasing treatment partner caregiver burden. Aggregate viral suppression rates significantly increased in the intervention (vs. control) clinic. Qualitative data from 14 clinic staff, 21 patients, and 16 treatment partners indicated that Mopati was viewed as effective. Providers said the guidance empowered them to be proactive in communicating about adherence; most reported using the guidance.

**Conclusion:**

This study shows preliminary support for the use of treatment partners in HIV care, and further evidence for interventions that leverage patients’ existing support. This research can inform ways to improve adherence to HIV treatment as well as the treatment of HIV-related comorbid conditions in lower-resource settings.

**Trial Registration:**

ClinicalTrials.gov Identifier: NCT04796610.

## Introduction

The leveraging of existing social relationships is a promising low-cost and scalable solution to ending the HIV epidemic. Social connectedness, close relationships, and social support have robust and large effects on health [1, 2], in part by motivating behavior change. Social support as an intervention for people living with HIV (PLWH) is written into multiple national HIV treatment guidelines in the form of treatment partners [3–5], or recommendations for PLWH to select informal caregivers who can help support their adherence when they start antiretroviral therapy (ART). In practice, patients are given little guidance about treatment partner selection, and treatment partners are not educated about how to support patients, and although immediate ART initiation following diagnosis has led to increased viral suppression rates [6], selection of treatment partners is now less likely prior to ART initiation [7].

Failure to engage treatment partners represents a missed opportunity, as PLWH who select treatment partners show increased adherence and viral suppression (mainly in observational designs, with mixed effects for randomized controlled trials) [8]. Treatment partners motivate adherence, improve psychosocial functioning, increase disclosure, and reduce stigma [9–11]. Prior research has shown that PLWH are more likely to be virally suppressed if they have treatment partners who support their holistic needs and well-being in addition to adherence [7]. In the same study, spouses and/or intimate relationship partners were most effective, likely because of their frequent close contact. Moreover, treatment partners expressed a desire to learn counseling skills to increase patients’ motivation.

Following self-determination theory, caregivers such as treatment partners motivate lasting behavior change when they support autonomous behavior regulation—in which patients freely choose to engage in behaviors—which increases intrinsic motivation [12, 13, 23]. In contrast, controlled behavior regulation, when individuals perform behaviors because they feel pressured, can undermine intrinsic motivation [14]. Similarly, in contrast to directive support that prescribes “correct” feelings and choices, peers’ non-directive support—acceptance of feelings and choices—is related to better mental health and health behaviors among patients [15, 16].

We used mixed methods to examine feasibility, acceptability, and preliminary effectiveness of an enhanced clinic-based treatment partner intervention in Botswana called Mopati (“one who accompanies another” in Setswana), which included standardized language for providers to guide patients on treatment partner selection and workshops to train treatment partners on providing non-directive support to patients using a non-confrontational, non-judgmental approach. Botswana has a high HIV prevalence (18.6% among those aged 15–49) [17], despite great strides toward the UNAIDS goal to end the HIV epidemic by 2030 [18]. Some interventions have been developed to help treatment partners support patients, but none with a healthcare provider or whole-clinic component [19–22]. Based on self-determination theory [23], we hypothesized that Mopati would increase treatment partners’ supportive behaviors, thus improving patient–treatment partner relationships. As a result of improved relationships, consistent with prior research [11], we hypothesized that patients’ intrinsic motivation and adherence self-efficacy would increase, and patients would exhibit higher adherence and viral suppression. We also expected that improved patient–treatment partner relationships would increase patients’ well-being and reduce treatment partners’ perceived caregiver burden.

## Methods

### Setting

This research took place in two infectious disease care clinics (IDCCs) in the Greater Gaborone District Health Management Team in Gaborone, Botswana. From a list of seven clinics in the region, the closest matched pair was selected based on caseload, staffing, viral suppression rates, sufficient geographical distance from each other (to avoid contamination), and receptivity to the study, as assessed in staff focus groups and clinic management discussions [7]. The clinics were randomly assigned to the intervention or standard of care.

### Stakeholder Engagement

The team presented the study to the standing community advisory board (CAB) of PLWH at one of the lead investigator’s institutions. The team met regularly with clinic management throughout the project and trained intervention clinic staff about a month prior to starting patient enrollment. Results were discussed with clinic staff.

### Participant Eligibility

Patients were eligible if they were 18 years old or older, diagnosed with HIV (at least 6 months prior), on ART for at least 6 months, and not virally suppressed, and ineligible if they had ART resistance (all confirmed with medical records). Treatment partners selected by patient participants were eligible if they were at least 18 years old. We prioritized recruiting patients who had treatment partners or who selected new treatment partners after receiving guidance from providers (described below). We allowed participation of patients who did not have a treatment partner or whose treatment partner could not participate, due to challenges in recruiting treatment partners during the COVID-19 pandemic (when in-country movement was restricted).

### Procedures

Patients were recruited by telephone by study staff, who received a list of potentially eligible patients from each clinic approximately monthly during recruitment (June 2021–March 2022). Eligible patients were encouraged to refer their treatment partner. Patients and treatment partners completed phone interviews at baseline and 3-month follow-up using survey software (REDCap version 12.4.0) from June 2021 to June 2022. Patients and treatment partners in the intervention clinic were asked to attend at least three sessions together. In-person intervention sessions, conducted with COVID-19 precautions, were scheduled approximately twice monthly (July 2021–June 2022). Sixty patients (30 per clinic) and 45 treatment partners (17 intervention, 28 control) were recruited. Participants were given incentives of 100 pula (~ $8.00) per survey. Participants who attended intervention sessions were given 30 pula (~ $2.00) for transport; in the few cases in which transport costs were higher, actual transport costs were provided. Participants provided verbal informed consent. Procedures were approved by the RAND Human Subjects Protection Committee (HSPC 2019-0253) and the University of Botswana Institutional Review Board (UBR/RES/IRB/BIO/146).

### Intervention and Standard of Care Control Description

#### Healthcare Provider Guidance

Drawing on our formative research [7], we developed a script for healthcare providers with standardized language for guiding all new and virally unsuppressed patients on treatment partner selection, and for guiding patients with a treatment partner on how to select an alternative treatment partner, if the patient expressed concerns about the support they were (or were not) receiving. The script incorporated autonomy-supportive elements from motivational interviewing (MI) [24], using a non-confrontational, non-judgmental approach that emphasized guiding without pressuring patients. All clinic staff received an approximately 2-h training in May 2021.

#### Treatment Partner Workshop

Treatment partner–patient dyads were encouraged to attend a workshop together on how to provide non-directive support using an MI-styled approach [24]. Each 1-h group session consisted of (1) basic HIV treatment education, (2) how MI strategies (open questions, reflective listening, affirming) can support patients’ motivation to adhere by conveying acceptance and compassion in a non-judgmental and non-confrontational manner, and (3) role-plays and examples of responding to challenging situations. Three core sessions were developed with different HIV information, MI skills, and role-plays. Sessions were run as open groups (in which new and returning participants could be mixed), to match how implementation might be feasibly done during future dissemination. Although the workshop focused on training treatment partners on how to support patients, patients could attend alone if their treatment partner was not available. Patients who attended alone were counseled on how to use MI strategies themselves and engage with their treatment partners to obtain support.

The master-level counselor received an 8-h group facilitation and MI skills training from an American clinical psychologist with MI training expertise who provided ongoing supervision. A research assistant observed each session and was trained to rate fidelity to key session content elements (not at all covered, somewhat covered, completely covered) and MI skills usage (1 (not at all) to 7 (very much)) [25].

#### Standard of Care (Control Clinic)

The standard of care included telling patients (primarily those who were unsuppressed) about the option of selecting a treatment partner (someone to whom they disclose their serostatus, who can support their HIV care) [7]. Unsuppressed patients were scheduled to visit the clinic monthly (more frequently than suppressed patients).

### Assessment

#### Sociodemographic Characteristics

Patients and treatment partners reported gender, age, marital status, education, employment status, time since HIV diagnosis, and income.

#### Patient Adherence

Patients reported the percentage of prescribed doses taken in the last month, which has been validated against HIV viral load [26]. We dichotomized adherence as a clinically significant score (80–100% vs. < 80%). Adherence self-efficacy was assessed with the item, “How confident are you that you can follow your antiretroviral medication dosing instructions exactly as prescribed by your doctor?” from 0 (“not confident at all”) to 10 (“completely confident”) dichotomized 0–9 vs. 10 because 79% of responses were 10 [27].

#### Patient Viral Load

Because intervention clinic providers were asked to use the treatment partner selection guidance with all new and unsuppressed patients, we obtained monthly aggregate, whole-clinic viral suppression rates over the intervention period (July 2021–June 2022). Note that we only obtained institutional review board approval to collect aggregate monthly percentages for viral suppression, not exact viral load values, for each clinic as a whole. Thus, viral suppression for clinic-level data was defined according to the Botswana HIV Clinical Care Guidelines at the time of the study (< 400 copies of HIV per ml of blood) [3].

We also obtained institutional review board approval to extract most recent, exact viral load values from individual patient participant medical records, for which we defined viral suppression as < 200 copies of HIV per ml of blood, consistent with U.S. Centers for Disease Control and Prevention (CDC) guidelines. However, many clinic assessments of viral load did not match the timing of the follow-up study assessments (e.g., they were measured during, before, or well after the intervention period, rather than immediately after). When we restricted participant viral load values to those that were assessed 90 to 180 days post-baseline (and after the last intervention session attended, for intervention participants), only 18 intervention and 16 control participants had eligible viral load values for the study.

#### Patient Autonomous Regulation for Adherence

Patients completed the autonomous motivation (*α* = 0.26) and controlled motivation (*α* = 0.72) subscales of the Treatment Self-Regulation Questionnaire [13], from which a Relative Autonomy Index (RAI) was derived (autonomous-controlled regulation subscale average). Because of the autonomous motivation subscale’s low alpha, we also derived the RAI with a two-item subscale, dropping one item that was negatively correlated; because results were similar, we report results for the full RAI.

#### Patient Well-being

The Internalized AIDS-Related Stigma Scale (average of items; *α* = 0.89) [28] and the PHQ-8 (a depression screener; *α* = 0.91) were used [29].

#### Dyadic Relationship Quality

Patient perceptions of directive (*α* = 0.72) and nondirective (*α* = 0.92) support from their treatment partner were assessed with subscales adapted from the Social Support Inventory [16]. Response options were 1 (“not at all typical”) to 5 (“very typical”).

#### Caregiver Burden

Treatment partners completed an adapted Caregiver Burden Inventory (0 (“never”) to 3 (“frequently or nearly always”)), with average scores for embarrassment/anger (*α* = 0.91), patient dependency (*α* = 0.62), and self-criticism (*α* = 0.85) [30]. Because the subscale scores had skewed distributions, we dichotomized them (0 vs. > 0).

### Healthcare Provider Survey at Intervention Site

From September 2021 to June 2022, study staff emailed and brought hard copies of an anonymous survey to all 14 clinic staff monthly to assess the extent to which they used the guidance with new and unsuppressed patients separately (0 (“not at all”) to 2 (“very much”)).

### Statistical Analysis

 Comparability between intervention and control groups at baseline was tested (Table 1). In an intention-to-treat analysis, linear regressions for continuous survey outcomes and logistic regressions for dichotomous survey outcomes were conducted, using each outcome’s baseline value and condition as predictors. For viral suppression, the only predictor was condition (because all patients were unsuppressed at baseline). In adjusted analyses, we used as covariates patient and treatment partner characteristics that significantly varied by clinic (at *p* < 0.05; patient age for patient outcomes, treatment partner income for treatment partner outcomes). Mean imputation was used to account for any missingness of baseline items (very low: 0–4 participants). Effect sizes were estimated following established statistical procedures [31].

**Table 1:** Patient and treatment partner baseline characteristics by clinic

**Baseline characteristic**^**a**^	**Overall**	**Intervention clinic**	**Control clinic**	**Intervention vs. control *p* value**^**b**^
**Age, *M* (SD)**				
Patient	40.5 (11.5)	37.0 (10.4)	43.8 (11.6)	*0.02*
Treatment partner	40.4 (10.0)	40.8 (11.2)	40.1 (9.5)	0.84
**Female, %**				
Patient	62.1	60.7	63.3	1.00
Treatment partner	75.6	70.6	78.6	0.72
**Dyadic gender category, %**				
Both female	44.4	41.2	46.4	0.83
Both male	0.0	0.0	0.0	
Patient is male; treatment partner is female	31.1	29.4	32.1	
Patient is female; treatment partner is male	24.4	29.4	21.4	
**Marital status: married or cohabitating, %**				
Patient	34.5	46.4	23.3	0.10
Treatment partner	35.6	41.2	32.1	0.75
**Secondary education, %**				
Patient	41.4	35.7	46.7	0.44
Treatment partner	60.0	47.1	67.9	0.22
**Employed, %**				
Patient	72.4	67.9	76.7	0.56
Treatment partner	64.4	47.1	75.0	0.11
**Formal full-time employment, %**				
Patient	36.2	39.3	33.3	0.79
Treatment partner	40.0	23.5	50.0	0.12
**No monthly income, %**				
Patient	22.8	25.9	20.0	0.75
Treatment partner	37.5	58.8	21.7	*0.02*
**Treatment partner is HIV positive, %**	44.4	47.1	42.9	1.00
**Patient adherence** (≥ 80% in the past month; self-report), **%**	89.5	85.7	93.1	0.42
**Time since HIV diagnosis, *M* (SD)**	9.4 (5.4)	8.3 (5.9)	10.4 (4.7)	0.14

^a^Marital status was assessed as *married or living with someone*, *in a relationship but living separately*, *single*, *never married*, *divorced or separated*, and *widowed*, and dichotomized at *married or living with someone* vs. all others. Education was assessed as *non-formal*, *primary*, *junior*, *senior*, *higher than secondary*, and *university level*, and dichotomized at *junior or lower* vs. *senior or higher*. Nature of employment was assessed as *occasional or casual*, *seasonal*, *formal wage (full-time)*, *formal wage (part-time)*, *self-employed in agriculture*, *self-employed making money full time*, and *self-employed making money part time*, and dichotomized at *formal wage employment (full-time)* vs. all others. Income was assessed as *no income*, *1–199 pula*, *200–499 pula*, *500–999 pula*, *1000–4999 pula*, *5000–10,000 pula*, and > *10,000 pula*, and dichotomized at *no income* vs. all others. Sample sizes for most characteristics were 58 patients and 45 treatment partners (total), 28 patients and 17 treatment partners (intervention), and 30 patients and 28 treatment partners (control), except for income (57 patients and 40 treatment partners), and adherence (57 patients)

^b^*p* values from Fisher’s exact test for binary characteristics, *t* test for age, and chi-square test for dyadic gender distribution. Significant *p*-values (< 0.05) are italicized

Clinics were compared on overall viral suppression rates using a repeated-measures regression with month, condition, and the month by condition interaction as predictors. The model included all 12 months of data, with month treated as linear. A second model, conducted as a sensitivity analysis, included only the first and last months of data, treating month as binary.

### Qualitative Assessment and Analysis

Intervention acceptability and feasibility were assessed qualitatively with two post-intervention focus groups of 14 intervention clinic staff (13 women, 1 man), and semi-structured interviews with 21 patients and 16 treatment partners. Focus groups were conducted by the study coordinator (a Motswana female with a master’s degree in counseling). Exit interviews were conducted by a male study nurse and a female research assistant (both Motswana). Transcripts were translated from Setswana to English as needed. A directed content approach was used for analysis, starting with constructs from prior acceptability and feasibility frameworks [32], allowing for new themes to emerge [33]. One investigator (female PhD social psychologist from the USA) and the coordinator independently reviewed all transcripts and together created codebooks. They independently double coded one focus group (50% of focus groups; 23 passages) and four interviews (12% of interviews; 72 passages), attaining good coder concordance (*K* = 0.84 for the focus group and *K* = 0.91 for interviews). Remaining data were coded using Dedoose software by the study coordinator; codes were reviewed by the investigator.

## Results

### Participants

**Fig. 1:**
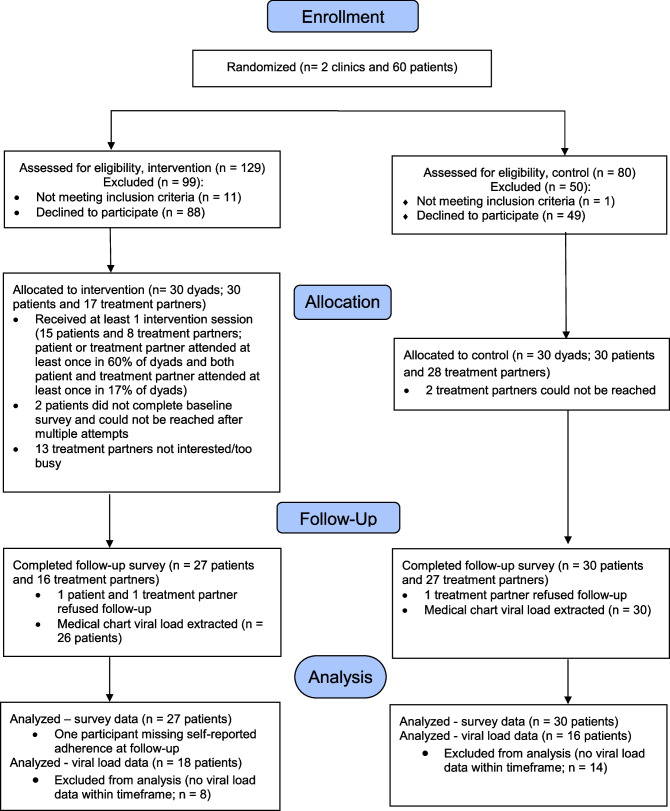
CONSORT patient flow diagram for Mopati pilot randomized controlled trial

Figure 1 shows the participant flow through the study, and Table 1 shows final sample characteristics. Sixty patients (30 per clinic) and 45 of their treatment partners (28 control, 17 intervention) were recruited. Thus, only 45 dyads of 60 possible dyads participated, because some patients did not have treatment partners, and some treatment partners chose not to participate. Among the 137 patients who were eligible but who did not participate, 29 were not interested, 8 were too busy, 5 were transferred to other clinics, 4 had disclosure issues, 2 had health issues, and 13 had treatment partners who were not interested. The remainder did not provide a reason. A total of 57 patients (95%; 27 intervention, 30 control) and 43 treatment partners (96%; 16 intervention, 27 control) were retained at follow-up. Twenty patients (67%) and 16 treatment partners (94%) in the intervention clinic completed a post-intervention exit interview.

### ART Adherence and Viral Suppression

#### Patient-level Data

In adjusted analyses, compared to control patients, in the intervention group, self-reported adherence significantly increased (with a large effect size), relative autonomous regulation for adherence significantly increased, and adherence self-efficacy marginally increased at follow-up (Table 2). The likelihood of viral suppression did not significantly differ between conditions.

**Table 2:** Effect of Mopati intervention on HIV-related and psychosocial outcomes

**Outcome**	**Intervention** ***M* (SD) or % (*n* = 27 patients and 16 treatment partners)**		**Control** ***M* (SD) or % (*n* = 30 patients and 27 treatment partners)**		***b* (SE) or OR (95% CI); Cohen’s *d*, *p* value (unadjusted)**	***b* (SE) or OR (95% CI); Cohen’s *d*, *p* value (adjusted)**
	**Baseline**	**Follow-up**	**Baseline**	**Follow-up**		
**HIV-related outcomes**						
≥ 80% of doses, past month (self-report), *n* = 57	85.2%	96.3%	93.0%	83.3%	5.9 (0.6–57.8); 0.98, 0.13	16.9 (1.2–233.4); 1.56, *0.03*
High adherence self-efficacy^a^, *n* = 57	74.1%	81.5%	83.3%	73.3%	2.2 (0.5–8.8); 0.42, 0.28	5.0 (0.9–28.0); 0.88, 0.07
Autonomous regulation for adherence^b^, *n* = 55	2.6 (1.7)	2.7 (1.3)	1.6 (1.5)	1.4 (2.0)	1.3 (0.5); 0.70, *0.02*	1.3 (0.5); 0.70, *0.02*
Viral suppression (< 200 copies), *n* = 31	0%	66.7%	0%	81.3%	0.5 (0.1–2.3); − 0.43, 0.34	0.6 (0.1–3.0); − 0.33, 0.49
**Patient well-being**, *n* = 57						
Internalized stigma	2.5 (1.5)	1.9 (1.3)	2.0 (1.2)	1.9 (1.0)	− 0.2 (0.3); − 0.18, 0.46	− 0.3 (0.3); − 0.29, 0.22
Depression symptoms	3.1 (5.3)	2.0 (3.3)	7.0 (6.5)	3.9 (3.3)	− 1.8 (0.9); − 0.53, 0.06	− 1.9 (1.0); − 0.54, 0.07
**Dyadic relationship**, *n* = 51						
Directive support	4.2 (1.1)	4.6 (0.9)	4.0 (1.2)	4.2 (1.2)	0.4 (0.3); 0.34, 0.24	0.4 (0.3); 0.40, 0.20
Non-directive support	4.4 (1.0)	4.9 (0.2)	4.6 (0.7)	4.5 (1.0)	0.4 (0.2); 0.51, 0.07	0.5 (0.2); 0.64, *0.03*
**Caregiver burden**, *n* = 43						
Any embarrassment/anger	37.5%	18.8%	37.0%	33.3%	0.5 (0.1–2.0); − 0.43, 0.31	0.3 (0.1–1.7); − 0.64, 0.18
Any dependency	37.5%	6.3%	63.0%	55.6%	0.1 (0.0–0.6); − 1.57, *0.01*	0.1 (0.0–0.7); − 1.48, *0.02*
Any self-criticism	87.5%	62.5%	88.9%	66.7%	0.8 (0.2–3.1); − 0.09, 0.80	1.7 (0.3–8.4); 0.29, 0.52

^a^Dichotomized as 10, extremely confident, vs. 0–9, less confident

^b^Because of the autonomous motivation subscale’s low reliability, we examined adjusted effects with a two-item subscale and results were similar (*b* (SE) = 1.3 (0.5), *p* = 0.02). Multivariate regressions on patient outcomes and treatment partner outcomes controlled for patient age and treatment partner income, respectively. Significant *p* values (< 0.05) are set in italics

#### Clinic-Level Data

**Fig. 2:**
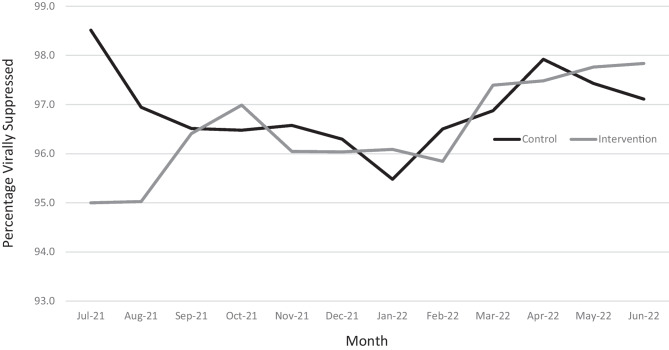
Aggregate clinic viral suppression during the intervention period for intervention and control clinics. A significant linear interaction effect over time (odds ratio (95% CI) = 1.06 (1.01–1.12), *p* = 0.03; Cohen’s *d* = 0.033 per month increase of 0.39 over 12 months) and a significant time by intervention condition interaction for the last month vs. first month (odds ratio (95% CI) = 4.69 (1.37–16.10), *p* = 0.01; Cohen’s *d* = 0.85) are shown. Data from 14,268 viral load assessments across the 12 months (monthly range 777–1434) are reflected

Although the control clinic started with a higher aggregate viral load suppression rate (Fig. 2), a significant linear intervention effect indicated that aggregate viral suppression rates increased in the intervention clinic and decreased in the control clinic over time (OR (95% CI) = 1.06 (1.01–1.12), *p* = 0.03; Cohen’s *d* = 0.033 per month increase of 0.39 over 12 months, a small-to-medium effect). The time by intervention condition interaction was significant for the last month (June 2022) compared to the first month (July 2021) (OR (95% CI) = 4.69 (1.34–16.10), *p* = 0.01; Cohen’s *d* = 0.85, a large effect).

### Psychosocial Outcomes

Intervention patients perceived significantly higher non-directive support from their treatment partners (and similar directive support) at follow-up compared to control patients (Table 2). Intervention patients endorsed marginally fewer depression symptoms at follow-up; internalized stigma did not significantly differ. Treatment partners in the intervention clinic reported reduced caregiver burden (significantly lower patient dependency) than did treatment partners in the control clinic, with a large effect size.

### Implementation (Provider Guidance)

Across the 14 providers, an average of 8.3 (3.9) surveys were completed monthly (range = 2–14). Ratings were high for new (*M* (SD) = 1.7 (0.5)) and unsuppressed (*M* (SD) = 1.8 (0.5)) patients during the participant enrolment period (August 2021–April 2022), with most patients likely exposed to the guidance. Ratings decreased post-enrolment (May–June 2022; *M* (SD) = 0.9 (0.5) for new patients and 1.1 (0.7) for unsuppressed patients). In focus groups, providers said the guidance empowered them to be proactive in communicating with patients about adherence and enlightened patients about the need to seek social support (Table 3). Providers said they used the guidance because they observed its effectiveness first-hand, with increased viral suppression rates and decreased overall clinic workload. Providers related that the guidance was easy to integrate into daily work responsibilities, as they were already talking with patients about adherence; the guidance gave them a strategy for approaching patients and starting the conversation comfortably. Providers noted barriers, including limited time. Providers discussed stigma and lack of HIV serostatus disclosure as key impediments to treatment partner selection. To improve implementation, providers suggested shortening the guidance and making it more accessible and engaging (e.g., digitized guidance for phones, using graphics and dialogue). They expressed that patients could be counseled about treatment partner selection in groups or queues. Providers suggested a refresher training (especially for addressing non-disclosure).

**Table 3:** Qualitative provider themes for standardized provider guidance (*n* = 14 healthcare providers over two focus groups)

**Theme**	**Healthcare provider exemplary quotes**
High acceptability and feasibility	“It helps us even when approaching patients… you do not just begin struggle to talk to them…Even when they ask questions you would know how to answer them.”; “As for me I think it didn’t [increase workload]. These are the things we do when a client comes, when we counsel them. It’s in our job description that we do it”
Patient barriers	*Stigma*: “Most of our clients still feel that the community is stigmatizing them, so it becomes so hard to ask them to share or to have someone to support them because they feel judged.” *Patients too busy*: “Our patients are always in a hurry…What they want is for them to be prescribed, given their medication and go back to work. So when you try to share other things with them, they will tell you that I have to go to work, my bosses are complaining”
Implementation recommendations	*Address patients in groups*: “Maybe once a week while they queue for registration there may be just one person that addresses them and informs them about the program and so those that are interested may ask the doctor for clarity. We can make it brief so that it doesn’t take much time because others stay alone or cannot read.” *Digitize guidance*: “Maybe making it digital, electronic? Because we are always on our phones. So that if we can have it, it will be easier for us to access it. Well, nowadays with papers, we are lazy, we are lazy to flip through papers.” *Add graphics and dialogue*: “You can improve it by using some pictures, people exchanging verbal communication”
Training recommendations	*Train all staff*: “All the staff could be spoken to so that all of us could know how to speak to patients even that one who is difficult… you could come train all of us even those who are volunteering.” *Conduct refresher training*: “We might have the knowledge and the practical part of it but we might struggle so maybe we need a refresher course”

### Implementation (Workshop)

Fifteen patients (50%) and eight treatment partners (47%) representing 60% of dyads attended at least one of the 22 sessions conducted. Nearly all fidelity ratings were high (*M* (SD) across sessions = 95.8% (6.2), “completely covered”; MI skills: *M* (SD) = 6.9 (0.1)). Patients, treatment partners, and providers perceived that the workshop was effective in improving adherence (Table 4). Patients and treatment partners said they enjoyed the workshop and learned about HIV, adherence, and communication skills. Reasons provided for non-attendance by patients included having work (*n* = 7), being out of town (*n* = 1), having childcare responsibilities (*n* = 1), and not being reachable by the study team during the intervention period (*n* = 1). Reasons provided by treatment partners included having work or childcare (*n* = 6), being out of town (*n* = 2), and moving (*n* = 1). Others who did not attend could not be reached. Providers discussed limited space and staffing as barriers. Patients and treatment partners suggested holding sessions during non-clinic hours. Providers asked for a refresher training, so existing staff could conduct workshops or support implementation by external facilitators.

**Table 4:** Qualitative themes about treatment partner workshop (from semi-structured interviews with 21 patients and 16 treatment partners, and focus groups with 14 healthcare providers)

**Theme**	**Patient quotes**	**Treatment partner quotes**	**Provider quotes**
High acceptability	*Improves communication*: “I have learnt how I can speak better with my husband without shouting at him and listening to him” (female, 40 years)	*Improves communication*: “[The workshops] educate treatment partners on how they can communicate with their patients in a way that can give them hope and encourage them not to lose hope” (female, 40 years, HIV-positive)	*Empowers providers and patients*: “[Mopati] was a very welcome development because it was going to empower us as healthcare providers, and it was going to empower our patients as well to manage their treatment”
High perceived effectiveness	*Improves adherence*: “I saw a change in my viral load. It went down. The viral load suppressed and now it has stuck to my mind that I must take meds on time. I no longer remember late and then do not take meds like I used to, you see? Yes, thinking that the time has passed, I will take the following day” (female, 35 years) *Reduces stigma*: It was helpful because in instances of romantic partners, it was always a problem when I had to tell them about my status. I would tell them but afterwards feel a little embarrassed. So, it really had motivated me and made me see that I don’t have a problem and I was just living in unnecessary embarrassment. (Male, 34 years)	*Improves adherence*: “I personally believe it was beneficial to them … those who attend will listen and learn its importance…during these sessions it is well explained that not taking medication properly is a drawback hence why it is mandatory to have the support of a treatment partner. That is why the sessions include treatment partners, so that if at all there are disagreements in the future between you and your treatment partner you may be able to remind them of what was taught during these classes and figure out everything together” (female, 45 years, HIV-negative) *Reduces stigma*: “It helps for us to accept each other. It helps so that we don’t suffer. We are sick but we still live” (female, 64 years, HIV-positive)	*Improves adherence*: “We are observing results…Ever since we have had Mopati, we are seeing an improvement. Some are suppressing. So, I confidently feel that way, confident that it is needed”
Well facilitated	“[The counselor] was very welcoming. Open to questions and she spoke to us tenderly without judging” (female, 40 years)	“She was explaining in detail and she was able to elaborate her points, so that people understand, while she was busy teaching” (female, 30 years, HIV-negative)	N/A
Feasibility issues	*Attendance barriers (e.g., work, busy, lives far)*: “I have a 07:30 to 17:00 job. So, it was not easy to get to the clinic during weekdays when I was needed. I was self-employed when you phoned me and asked me if I will be able to attend but I then got a job. It then became hard to come for the sessions” (female, 36 years)	*Attendance barriers (e.g., work, busy, lives far)*: “There was no one to do my duties on my behalf at work” (male, 54 years, HIV-positive)	*Implementation barriers (staffing, space)*: “If we have to do once a week maybe the staff would be enough but if daily then we’ll be short-staffed.” *Workload not a barrier*: “I don’t think it will increase the workload because it also benefits us…It makes our work easier because they sensitize people to give them information and empowerment”
Implementation recommendations	*Provide handouts*: “This is a very good initiative and it is advisable that you continue with this and help Batswana. I could see HIV decreasing in the future…Write it down somewhere and give them those handout pages to read and take notes” (male, 64 years)	*Provide handouts*: “Pamphlets…she must come with some tangible thing so that when I am home I start reading A,B,C the next time when I come back again it is me who is supposed to be telling them that when I went home I learnt this and that through reading” (female, 44 years, HIV-negative)	*Conduct provider training*: “The staff is enough… Everyone here could go through training, that way wherever the client goes, the helper will have knowledge on how to help them”

## Discussion

A pilot test of Mopati, a multi-level treatment partner intervention, showed mostly medium effect sizes on adherence and improved secondary outcomes (e.g., patient–treatment partner relationships, adherence self-efficacy) that are key predictors of adherence [34, 35]. Moreover, we found improved viral suppression rates over time in the intervention clinic. Intervention clinic suppression rates increased while control clinic rates decreased from July to August 2021, suggesting that the intervention enhanced healthcare system resilience during the pandemic, as there was a surge in COVID-19 cases during the same time period.

Providers reported frequently using the guidance with patients who were starting ART and patients who were not suppressed. Providers, as well as patients and treatment partners who attended workshop sessions, found the intervention to be acceptable. Nevertheless, barriers to in-person attendance include work schedules, limited feasibility, and overall acceptability. Willingness to attend in-person sessions may have been dampened by the COVID-19 pandemic, during which all study activities occurred. Alternative modes of intervention delivery, such as telephone and online sessions, might have yielded higher participation.

Our results are consistent with self-determination theory and indicate a need for interventions that support intrinsic motivation for adherence [12], not only for HIV but also for other chronic and comorbid conditions. Older PLWH are more likely to have co-morbid chronic conditions [36], for which treatment partners may be a low-resource effective option for bolstering support and adherence. However, of the few tests of treatment partner interventions, most focus on treatment partner training (e.g., directly observed therapy) [8] and have not engaged healthcare providers, patients, and treatment partners.

Limitations include the small sample size in two clinics, a high proportion of participants who declined the study and who did not participate in the intervention, limited retention, short-term follow-up, and use of self-reported adherence, which may be an overestimate [37]. To determine whether Mopati should be implemented for population-level impact, a cluster randomized controlled trial with long-term follow-up and a larger sample size is needed, as well as precise assessment of viral load through blood draws. Future studies could also assess required doses of different intervention elements, examine differential effects for sero-discordant dyads, and test for beneficial effects on treatment partners’ own health outcomes. Overall, the present study indicates further evidence for interventions that leverage patients’ support and has relevance for HIV healthcare as well as the management of HIV-related comorbidities.

## Declarations

### Data Transparency

All authors ensure that the data support the published claims and comply with field standards.

## References

[1] Holt-Lunstad J, Robles TF, Sbarra DA. Advancing social connection as a public health priority in the United States. Am Psychol. 2017;72(6):517. 28880099 10.1037/amp0000103 PMC5598785

[2] Shor E, Roelfs DJ, Yogev T. The strength of family ties: a meta-analysis and meta-regression of self-reported social support and mortality. Soc Networks. 2013;35(4):626–38.

[3] Republic of Botswana. Handbook of the Botswana 2016 integrated HIV clinical care guidelines. Botswana Ministry of Health 2016.

[4] Republic of Uganda. Consolidated guidelines for prevention and treatment of HIV in Uganda. Kampala, Uganda: Ministry of Health; 2016.

[5] Republic of South Africa. Adherence guidelines for HIV, TB, and NCDs. Department of Health, Pretoria. 2016. https://www.knowledgehub.org.za/elibrary/adherence-guidelines-hiv-tb-and-ncds. Accessed 16 Feb 2022.

[6] Havlir D, Lockman S, Ayles H, et al. What do the universal test and treat trials tell us about the path to HIV epidemic control? J Int AIDS Soc. 2020;23(2): e25455. 32091179 10.1002/jia2.25455 PMC7038879

[7] Bogart LM, Phaladze N, Kgotlaetsile K, Goggin K, Mosepele M. Qualitative evaluation of treatment partners for people with HIV in Botswana: Current healthcare provider practices and recommendations for improvement. Community Health Equity Research and Policy. In Press. 2023. 10.1177/2752535X231225809 PMC11219526 38171536

[8] Nyoni T, Sallah YH, Okumu M, Byansi W, Lipsey K, Small E. The effectiveness of treatment supporter interventions in antiretroviral treatment adherence in sub-Saharan Africa: a systematic review and meta-analysis. AIDS Care. 2020;32(Suppl2):214–27. 32196385 10.1080/09540121.2020.1742870

[9] Nachega JB, Knowlton AR, Deluca A, et al. Treatment supporter to improve adherence to antiretroviral therapy in HIV-infected South African adults: a qualitative study. J Acquir Immune Defic Syndr. 2006;43:S127–33. 17133196 10.1097/01.qai.0000248349.25630.3d

[10] Duwell MM, Knowlton AR, Nachega JB, et al. Patient-nominated, community-based HIV treatment supporters: patient perspectives, feasibility, challenges, and factors for success in HIV-infected South African adults. AIDS Patient Care STDS. 2013;27(2):96–102. 23373664 10.1089/apc.2012.0348 PMC3565551

[11] O’Laughlin KN, Wyatt MA, Kaaya S, Bangsberg DR, Ware NC. How treatment partners help: social analysis of an African adherence support intervention. AIDS Behav. 2012;16(5):1308–15. 21947835 10.1007/s10461-011-0038-4 PMC3354325

[12] Ryan RM, Patrick H, Deci EL, Williams GC. Facilitating health behaviour change and its maintenance: interventions based on self-determination theory. Eur Heatlh Psychol. 2008;10(1):2–5.

[13] Williams GC, Rodin GC, Ryan RM, Grolnick WS, Deci EL. Autonomous regulation and long-term medication adherence in adult outpatients. Health Psychol. 1998;17(3):269. 9619477 10.1037//0278-6133.17.3.269

[14] Stephens MA, Franks MM, Rook KS, Iida M, Hemphill RC, Salem JK. Spouses’ attempts to regulate day-to-day dietary adherence among patients with type 2 diabetes. Health Psychol. 2013;32(10):1029–37. 10.1037/a0030018. 23025302 10.1037/a0030018

[15] Kowitt SD, Ayala GX, Cherrington AL, et al. Examining the support peer supporters provide using structural equation modeling: nondirective and directive support in diabetes management. Ann Behav Med. 2017;51(6):810–21. 10.1007/s12160-017-9904-2. 28417438 10.1007/s12160-017-9904-2 PMC9574889

[16] Stewart DW, Gabriele JM, Fisher EB. Directive support, nondirective support, and health behaviors in a community sample. J Behav Med. 2012;35(5):492–9. 10.1007/s10865-011-9377-x. 21877174 10.1007/s10865-011-9377-x

[17] UNAIDS. Country factsheets: Botswana 2021. 2022. https://www.unaids.org/en/regionscountries/countries/botswana. Accessed 10 Oct 2022.

[18] Mine M, Stafford K, Laws RL, et al. Botswana achieved the Joint United Nations Programme on HIV/AIDS (UNAIDS) 95–95–95 targets: results from the Fifth Botswana HIV/AIDS Impact Survey (BAIS V), 2021. AIDS 2022; Montreal, Canada: International AIDS Society. 2022.

[19] Gross R, Zheng L, Rosa AL, et al. Partner-based adherence intervention for second-line antiretroviral therapy (ACTG A5234): a multinational randomised trial. The Lancet HIV. 2015;2(1):e12–9. 26424232 10.1016/S2352-3018(14)00007-1 PMC4313760

[20] Mugusi F, Mugusi S, Bakari M, et al. Enhancing adherence to antiretroviral therapy at the HIV clinic in resource constrained countries; the Tanzanian experience. Trop Med Int Health. 2009;14(10):1226–32. 19732408 10.1111/j.1365-3156.2009.02359.x

[21] Nakigozi G, Makumbi FE, Bwanika JB, et al. Impact of patient-selected care buddies on adherence to HIV care, disease progression, and conduct of daily life among pre-antiretroviral HIV-infected patients in Rakai, Uganda: a randomized controlled trial. J Acquir Immune Defic Syndr. 2015;70(1):75–82. 10.1097/qai.0000000000000710. 26039929 10.1097/QAI.0000000000000710 PMC4556592

[22] Simoni JM, Pantalone DW, Plummer MD, Huang B. A randomized controlled trial of a peer support intervention targeting antiretroviral medication adherence and depressive symptomatology in HIV-positive men and women. Health Psychol. 2007;26(4):488–95. 10.1037/0278-6133.26.4.488. 17605569 10.1037/0278-6133.26.4.488 PMC4044097

[23] Ryan RM, Deci EL. Self-determination theory and the facilitation of intrinsic motivation, social development, and well-being. Am Psychol. 2000;55(1):68. 11392867 10.1037//0003-066x.55.1.68

[24] Miller WR, Rollnick S. Motivational interviewing: helping people change. 3rd ed. New York: Guilford Press; 2013.

[25] Goggin K, Gerkovich MM, Williams KB, et al. A randomized controlled trial examining the efficacy of motivational counseling with observed therapy for antiretroviral therapy adherence. AIDS Behav. 2013;17(6):1992–2001. 23568228 10.1007/s10461-013-0467-3 PMC3672512

[26] Simoni JM, Kurth AE, Pearson CR, Pantalone DW, Merrill JO, Frick PA. Self-report measures of antiretroviral therapy adherence: a review with recommendations for HIV research and clinical management. AIDS Behav. 2006;10(3):227–45. 16783535 10.1007/s10461-006-9078-6 PMC4083461

[27] Chesney MA, Ickovics J, Chambers D, et al. Self-reported adherence to antiretroviral medications among participants in HIV clinical trials: the AACTG adherence instruments. AIDS Care. 2000;12(3):255–66. 10928201 10.1080/09540120050042891

[28] Kalichman SC, Simbayi LC, Cloete A, Mthembu PP, Mkhonta RN, Ginindza T. Measuring AIDS stigmas in people living with HIV/AIDS: the Internalized AIDS-Related Stigma Scale. AIDS Care. 2009;21(1):87–93. 10.1080/09540120802032627. 19085224 10.1080/09540120802032627

[29] Kroenke K, Strine TW, Spitzer RL, Williams JB, Berry JT, Mokdad AH. The PHQ-8 as a measure of current depression in the general population. J Affect Disord. 2009;114(1–3):163–73. 18752852 10.1016/j.jad.2008.06.026

[30] Flynn Longmire CV, Knight BG. Confirmatory factor analysis of a brief version of the Zarit Burden Interview in Black and White dementia caregivers. Gerontologist. 2011;51(4):453–62. 21402646 10.1093/geront/gnr011 PMC3146806

[31] Chinn S. A simple method for converting an odds ratio to effect size for use in meta-analysis. Stat Med. 2000;19(22):3127–31. 11113947 10.1002/1097-0258(20001130)19:22<3127::aid-sim784>3.0.co;2-m

[32] Proctor E, Silmere H, Raghavan R, et al. Outcomes for implementation research: conceptual distinctions, measurement challenges, and research agenda. Adm Policy Ment Health. 2011;38(2):65–76. 10.1007/s10488-010-0319-7. 20957426 10.1007/s10488-010-0319-7 PMC3068522

[33] Hsieh H-F, Shannon SE. Three approaches to qualitative content analysis. Qual Health Res. 2005;15(9):1277–88. 16204405 10.1177/1049732305276687

[34] Johnson MO, Neilands TB, Dilworth SE, Morin SF, Remien RH, Chesney MA. The role of self-efficacy in HIV treatment adherence: validation of the HIV Treatment Adherence Self-Efficacy Scale (HIV-ASES). J Behav Med. 2007;30(5):359–70. 17588200 10.1007/s10865-007-9118-3 PMC2423379

[35] Gonzalez JS, Batchelder AW, Psaros C, Safren SA. Depression and HIV/AIDS treatment nonadherence: a review and meta-analysis. J Acquir Immune Defic Syndr. 2011;58(2). 10.1097/QAI.0b013e31822d490a PMC3858003 21857529

[36] Feinstein MJ, Hsue PY, Benjamin LA, et al. Characteristics, prevention, and management of cardiovascular disease in people living with HIV: a scientific statement from the American Heart Association. Circulation. 2019;140(2):e98–124. 31154814 10.1161/CIR.0000000000000695 PMC7993364

[37] Arnsten JH, Demas PA, Farzadegan H, et al. Antiretroviral therapy adherence and viral suppression in HIV-infected drug users: comparison of self-report and electronic monitoring. Clin Infect Dis. 2001;33(8):1417–23. 11550118 10.1086/323201 PMC2692641

