# Author Correction: Pilot Test of Mopati, a Multi-Level Adherence Intervention for People Living with HIV and Their Treatment Partners in Botswana

**DOI:** 10.1007/s12529-023-10243-5

**Published:** 2023-12-13

**Authors:** Laura M. Bogart, Nthabiseng Phaladze, Keonayang Kgotlaetsile, David J. Klein, Kathy Goggin, Mosepele Mosepele

**Affiliations:** 1 RAND Corporation, 1776 Main Street, P.O. Box 2138, Santa Monica, CA 90407-2138 USA; 2 Charles R. Drew University of Medicine and Science, Los Angeles, CA USA; 3 University of Botswana, Gaborone, Botswana; 4 Health Services and Outcomes Research, Children’s Mercy Kansas City, Kansas City, MO USA; 5 Kansas City Schools of Medicine and Pharmacy, University of Missouri, Kansas City, MO USA; 6 Botswana-Harvard AIDS Institute Partnership, Gaborone, Botswana

**Author Correction: International Journal of Behavioral Medicine** **https://doi.org/10.1007/s12529-023-10233-7**

The original online version of this article was revised: This submission was subject to non-standard Copyright. The usual Copyright line was replaced by the following:

© RAND Corporation and The Author(s) 2023.

